# Second Courses of Transcranial Magnetic Stimulation (TMS) in Major Depressive Episodes for Initial Responders and Non-Responders

**DOI:** 10.21315/mjms2019.26.3.8

**Published:** 2019-06-28

**Authors:** Saxby Pridmore, Sheila Erger, Tamara May

**Affiliations:** 1 TMS Department, Saint Helen’s Hospital, Hobart, Tasmania, Australia; 2 School of Medicine, University of Tasmania, Hobart, Tasmania, Australia; 3 School of Psychology, Deakin University, Burwood, Victoria, Australia

**Keywords:** transcranial magnetic stimulation, depression, neurostimulation

## Abstract

**Background:**

Transcranial Magnetic Stimulation (TMS) is effective in major depressive episodes (MDE). However, MDE may follow a chronic, relapsing course, and some individuals may not satisfactorily respond to a first course of TMS.

**Objective:**

To investigate the outcome of second courses of TMS.

**Method:**

A naturalistic investigation—we prospectively studied 30 MDE in-patients and routinely collected information, including pre- and post-treatment with Six-item Hamilton Depression Rating Scale (HAMD6), a six-item Visual Analogue Scale (VAS6) and the Clinical Global Impression-Severity (CGI-S). Two categories of patients were considered: i) those who had remitted with a first course, but relapsed, and ii) those who had not remitted with the first course.

**Results:**

Thirty individuals received a second TMS course. The mean time to the second course was 27.5 weeks. Based on the HAMD6, 26 (87%) achieved remission after the first course, and 22 (73%) achieved remission after the second course. Furthermore, based on the HAMD6 results, of the four patients who did not achieve remission with a first course, three (75%) did so with a second course.

**Conclusion:**

In MDE, a second course of TMS is likely to help those who remitted to a first course and then relapsed, as well as those who did not achieve remission with a first course.

## Introduction

### Second Courses of TMS for Major Depressive Episodes

Major depressive episodes (MDE) are painful, disabling and common. Remission is difficult to achieve (1) and relapse frequently occurs (2). Transcranial magnetic stimulation (TMS) is an effective treatment for acute MDE (3). However, the clinical gains from active treatment may be lost over time.

Senova et al. (4) performed a meta-analysis regarding the durability of the TMS-induced response and found 52.9% lost response during the first six months post treatment. For comparison, Jelovac et al. (5) performed a meta-analysis on ECT-induced remission and found 37.7% relapsed during the first six months. However, the higher Senova et al. (4) figure refers to the loss of response, while the lower Jelovac et al. (5) figure refers to the greater change of relapse.

There are reports of MDE patients who have experienced a beneficial outcome from TMS and then relapsed and received a further course. Dannon et al. (6) reported on four patients who had ‘completed a successful TMS course previously’ but had relapsed and received a second course—for three patients, the second courses were as ‘equally effective’ as the first; however, for the fourth patient, the second course was less beneficial. Fitzgerald et al. (7) reported the ‘real world’ responses of 19 patients who had received from two to five TMS courses. In response to the second treatment course, using the Beck Depression Inventory (BDI), 12 patients achieved a > 50% reduction, five achieved a 25%–50% reduction and two did not change. Janicak et al. (8) followed 103 patients, and further TMS was prescribed for ‘symptom breakthrough’, which was defined as the loss of least one point on the Clinical Global Impression-Severity (CGI-S) for two weeks. They found 32 of the 36 patients (89%) benefited from the reintroduction of TMS.

Philip et al. (9) studied medication-free patients over one year. The reintroduction of TMS was considered a success when the 17-item Hamilton Depression Rating Scale (HAMD17) score reached the total that had been achieved with the initial acute treatment—this occurred for 14 of 18 patients (78%) in one group and 17 of 27 patients (63%) in another. Kelly et al. (10) found eight of 10 patients (80%) responded to a second course of TMS. Sackeim (11) concluded that reintroduction of TMS is unsuccessful in a ‘substantial proportion’ (approximately 30%) of cases.

In clinical practice, second courses of TMS may also be provided when first courses have failed to achieve a favourable outcome (response or remission). Following such failure, it is common for patients to receive a range of other options, and with a continued lack of satisfaction, to return to TMS hoping for a better outcome with a second course. To the best of our knowledge, there is no published account examining the outcomes of second courses when a first course has been unsuccessful.

We investigated the outcomes of a second course of TMS on the mood of patients with a history of MDE. Most of these patients had benefited from a first course; however, this benefit had been lost over time. There were also a few patients who had gained little from the first course but who came to a second course in hopes of a better outcome.

## Method

### Participants

We provide TMS in a private hospital in a state capital in Australia. There is no government funding for TMS in Australia; consequently, in our region, patients (privately insured) are generally admitted to the hospital to receive TMS (this arrangement enables costs to be recouped). Our unit has an academic attachment, and the Melbourne Clinic Ethics Committee approved this study. TMS is provided at the request of the treating psychiatrist, provided the known contraindications can be excluded and the DSM-V MDE diagnostic criteria are satisfied. This was a naturalistic study, and it was prospective in that we set a goal of recruiting 30 patients and accepted every patient who met the MDE inclusion criteria. A sample size of *n* = 30 provided the ability to detect a small to medium effect size (*f* = 0.22; using G*Power programme version 3, with the power [1 – β] set at 0.80 and α = 05, two-tailed), which was expected to be sufficient given the prior research findings regarding the medium to large effects of TMS (12). Patients remained under the care of their treating psychiatrist and continued to receive any prescribed medication.

### Procedure

The course included 20 daily TMS sessions over 22 days, with a two-day rest period after 10 treatments. Stimulation was applied at the left dorsolateral prefrontal cortex at 110% MT, 10 Hz, 4 s trains, 26 s inter-train interval and 75 trains (3000 pulses) per day. The prospective data collection commenced in January 2017 and was completed two years later.

### Measures

Pre- and post-treatment, we routinely collected: i) the objective six-item Hamilton Depression Rating Scale (HAMD6) (13) as the primary outcome measure, ii) the objective Clinical Global Impression-Severity (CGI-S) (14) and 3) a subjective six-item visual analogue scale (VAS6) (15), which is designed to complement the HAMD6. Earlier, we had found the VAS6 was a valid measure of the core aspects of depression measured by the HAMD6 (16).

The VAS6 anchor points are placed at either end of 10 cm lines: No depression–Worst possible depression; Activities give normal pleasure–Activities give no pleasure; No physical health concerns–Extreme physical health concerns; No feelings of guilt–Extreme feelings of guilt; Not anxious–Most anxious possible. The sixth HAMD6 item is an observational assessment of ‘retardation’ as a ‘best-fit’ match with the subjective anchor points we chose: No concentration problems–Most possible concentrations possible.

### Data Analysis

Repeated measures ANOVA was used to explore changes in the HAMD6, CGI-S and VAS6 over the four time points (pre-post the first treatment and pre-post the second treatment). Post-hoc *t*-tests were used to explore differences between pairs of scores—first course pre versus post, second course pre versus post, first course pre versus second pre and first course post versus second post. We used Bonferroni corrections of 0.0125 (0.05/4) as the significance level for post-hoc tests. Repeated measures ANCOVA was used in the same fashion, with the addition of the number of weeks between treatments used as a covariate.

We then explored the categories of remission and relapse on the HAMD6 and CGI-S by reporting the proportions of participants in each category. ‘Remission’ has been operationalised as an HAMD6 score of ≤ 4 (17) and a post-treatment CGI-S score of 2 or less (18). ‘Relapse’ is operationalised as a HAMD6 score of > 7 (16). The HAMD6 score of > 4 to 7 is termed ‘partial remission’ (18). ‘Non-remission’ was operationalised as a CGI-S score > 2.

## Results

Over the two-year period, we provided 150 courses to 120 individuals with a mean age of 44.1 years (SD = 15.6, range 18–88 years), and the group included 25 males (21%). Thirty individuals (25% of the index cases), a group whose mean age was 48.0 (SD = 16.6; range 20–76 years) and that included five males (17%), received a second course of TMS. The mean time between the first and second course was 27.5 weeks (SD = 16.7, range 9–69 weeks). One third of the patients reported occasional mild, temporary headaches; there were no other side effects.

Total scores were significantly reduced following each course across the HAMD6, CGI-S and VAS6 (Table 1). For the HAMD6, repeated measures ANOVA showed there was a significant main effect of time: *f*(3,87) = 141.5, *P* < 0.001, χ^2^ = 0.83. Post-hoc tests using paired samples *t*-tests showed a significant decrease in HAMD6 total scores before and after each treatment (*P* < 0.001). There was no difference in HAMD6 scores before the first and second course (*P* = 0.267) and no difference in first and second course post HAMD6 scores (*P* = 0.097). The number of weeks between the first and second course was used as a covariate to determine if it impacted the pre/post scores for the second course. The weeks had no impact: *f*(1,28) = 1.54, *P* < 0.23, χ^2^ = 0.05.

We repeated the above for the CGI-S scores with similar findings: post scores had decreased significantly for each treatment course, and there was no difference between pre scores for the first course. The second course post score was higher than the first course post score: *t*(29) = −2.1, *P* = 0.043, which was not statistically significant based on the corrected significance level. The number of weeks between treatments had no impact. The VAS6 scores were similar to the above; the second course post score was higher than the first course post score: *t*(29) = −2.5, *P* = 0.017; however, this was not statistically significant based on the corrected significance level.

### HAMD6 Categories

The HAMD6 categories of remission, partial remission and relapse were explored (Table 2). Before the first course, all 30 individuals were in relapse. After the first course, 26 (87%) were in remission, two (7%) in partial remission and two (7%) remained in relapse. The average weeks between the treatments was 26.2 (SD = 15.9) for those who achieved remission (*n* = 26) and 35.8 (SD = 21.8) for those who did not (*n* = 4).

Before commencing the second course, 29 (96%) individuals were in relapse, and one was in partial remission. After the second course, 22 (73%) achieved a second remission, 3 (10%) were in partial remission and five (17%) were in relapse.

Of the 26 individuals who had achieved remission following the first course of treatment, following the second course, 19 (73%) of them achieved remission, three achieved partial remission and four were in relapse. Of the two participants in partial remission following the first course, following the second course, one achieved remission and one remained in relapse. Of the two participants who had remained in relapse after the first course, following the second course, both achieved remission. Thus, three (75%) of those who had not achieved remission following the first course did so following the second.

### CGI-S Categories

The CGI-S categories of remission and non-remission were also explored. For the first course, 100% were in non-remission before treatment and 27 (90%) moved into remission with treatment. For the second course, 100% were in non-remission before treatment and 20 (67%) moved into remission with treatment.

Of the 27 individuals who had achieved remission following the first course, following the second course, 17 (63%) achieved remission. Of the three who had not achieved remission following the first course, all three achieved remission following the second course (Table 3).

## Discussion and Conclusion

This study aimed to understand the outcomes of a second course of acute TMS for MDE.

Thirty individuals underwent a second course of TMS. Using our primary outcome measure (HAMD6), after their first course, 26 of the 30 (87%) achieved remission—of these 26, 19 (73%) achieved remission after the second course. This suggests that certain feature/s of an individual and/or his/her disorder, separately or in concert, determine the likelihood of the individual-disorder combination responding to TMS and that such feature/s are relatively stable.

However, not all patients responded to the first course. We also studied the clinical outcome to a second course of TMS supplied to four people who had not achieved full remission with a first course. These patients had already failed to respond to medication and psychotherapy; thus, they were patients with highly resistant depression. Three (75%) achieved a remission, while one did not change. Theoretically, this could suggest that certain feature/s of these individuals and/or their disorders had changed over the intervening period (an average of 36 weeks), such that the second course produced the desired effect. Practically, this shows that a second course of TMS is worth further trials, even in severe cases that have not responded to an initial course. We do not know of this observation (that a second course may be successful when an initial course has failed) having been made previously in the literature; however, it is a known clinical practice.

The findings of a 73% remission rate following the second course of acute TMS was consistent with the findings of earlier authors. Sackeim (11) estimated that the re-introduction of TMS may produce remission in approximately 70% of individuals.

The limitations of this study include that it was neither placebo controlled nor blinded and that our patients continued to receive antidepressant medication and were hospitalised during treatment. Fitzgerald et al. (7) pointed out the difficulty of randomising and blinding studies when patients have experienced active treatment. All these patients were taking at least one anti-depressant, some were taking more than one anti-depressant and about half were also taking a mood stabiliser, anti-psychotic or benzodiazepine.

Evidence suggests that about a third of those who achieve remission will need further treatment in the first post-treatment year (9, 10, 20). Thus, relapse is a real-world event, and effective treatment options are needed.

The outcomes of second courses of TMS generally suggest the likelihood of a second remission. However, these studies have used a range of different tools and methods. Fitzgerald et al. (7) did not use formal criteria to define relapse, and Demirtas-Tatlidede et al. (21) considered response but not remission. Janicak et al. (8) did not measure subjective experience and employed a ‘symptom breakthrough’ defined using the CGI-S. Dunner et al. (20) reported the proportion of patients who received ‘reintroduction’ TMS (36.2%); however, they did not provide specific details of the outcome. Philip et al. (9) provided second TMS courses of less than 20 treatments. Kelly et al. (10) did not use an objective measure and defined both response and remission using unconventional percentage reductions in BDI scores. These accounts of second TMS courses are generally components of larger studies that address many aspects of the treatment of MDD with TMS. We have approached the question prospectively, using standardised tools, with a view to further extending understanding.

The mean weeks between the first and second treatment was 27.5 overall, 26 weeks for those who achieved remission after the first course (*n* = 26) and 36 weeks for those who did not (*n* = 4). There is a possible explanation for this longer period between treatments for those who did not achieve remission after the first course: when patients achieved a good response to the first treatment, deterioration took place, and the treating psychiatrists were predisposed to arrange a further TMS course. However, when the initial treatment provided poor results, other things were presumably tried, and the patients came back only after unsuccessful trials of other potentially therapeutic agents.

We chose the visual analogue scale as our means of quantifying subjective experience because it is a long-established method that has been usefully applied in a range of fields, and it provides information about an experience the instant it is completed (rather than over recent days). A possible disadvantage of the VAS is that the unbroken continuum scoring system is unfamiliar to most subjects and may cause difficulties. We will consider a Likert scale for future studies. There was significant improvement in subjective symptoms; however, the first course post score was significantly lower than the second course post score (suggesting less subjective benefit from the second course). This may reflect reality. Another consideration is the unfamiliar continuum scoring method may have introduced possible inaccuracies. Furthermore, when suffering patients (who have been disappointed by multiple other treatments) experience a positive outcome to their first course of TMS, they are usually surprised and highly delighted—it is quite possible they develop unrealistic expectations for the second course. Nevertheless, consistent with objective scores, the subjective mood improved significantly with both first and second TMS courses.

We have demonstrated that for people who regularly relapse within a few weeks of achieving remission, scheduled short courses over a few days at monthly intervals may be useful in preventing complete relapse (12, 16). In the current study, we are considering a different group—the patients in the current study were less prone to extremely rapid relapse.

We found that of the four patients who did not respond to a first course of TMS, three responded to a second course. While these numbers are small, they show a second course (following a failed course) may be beneficial. Our plan is to systematically explore this phenomenon.

## Figures and Tables

**Table 1:** Means and standard deviations for the pre-post scores (*n* = 30)

Time point	First course pre mean (SD)	First course post mean (SD)	Second course pre mean (SD)	Second course post mean (SD)	Significant differences
HAMD6 total score	11.1 (1.9)	3.0 (1.8)	10.4 (2.4)	3.8 (2.2)	First course pre > post* Second course pre > post*
CGI-severity	4.5 (0.6)	1.8 (0.7)	4.2 (0.7)	2.2 (0.8)	First course pre > post* Second course pre > post*
VAS total score	38.5 (7.9)	15.3 (9.5)	38.1 (8.0)	20.3 (9.3)	First course pre > post* Second course pre > post*

* *P* < 0.001

**Table 2:** Post treatment HAMD6 categories for first and second course of TMS

	First course (post-treatment)				

		Remission	Partial remission	Relapse	Total
Second course (post-treatment)	Remission	19	1	2	22
	Partial remission	3	0	0	3
	Relapse	4	1	0	5
	Total	26	2	2	30

**Table 3:** Post treatment CGI-Severity categories for first and second courses of TMS

	First course (post-treatment)			

		Remission	Relapse	Total
Second course (post-treatment)	Remission	17	3	20
	Relapse	10	0	10
	Total	27	3	30
